# The First Reported Case of Neurotrophic Tyrosine Receptor Kinase Fusion-Positive Thymoma Treated Successfully With Entrectinib

**DOI:** 10.7759/cureus.20588

**Published:** 2021-12-21

**Authors:** Hassan Salame, Remy Mckey, Mohamad Ballout, Wajih Saad

**Affiliations:** 1 Internal Medicine, Lebanese University Faculty of Medicine, Beirut, LBN; 2 Cardiology, Hospital Center Agglomeration De Nevers, Nevers, FRA; 3 Oncology, Al Zahraa Hospital University Medical Center, Beirut, LBN

**Keywords:** thymoma, antineoplastic agents, ntrk receptor, trka receptor, protein kinase inhibitors, whole genome sequencing, oncogene proteins fusion, entrectinib

## Abstract

We present the first reported case of stage 4 thymoma with pleural metastases that was found to be driven by the neurotrophic tyrosine receptor kinase (NTRK)-fusion gene. The patient was started on chemotherapy but it was discontinued due to intolerable side effects. Alternative options in such patients with rare diseases are limited; in fact, many concerns exist regarding the safety and efficacy of newly approved agents for the treatment of advanced thymomas, such as pembrolizumab and sunitinib. Due to NTRK-fusion gene positivity, entrectinib, a novel NTRK-fusion inhibitor, was then initiated. This drug has shown an objective response of 57% in treating NTRK fusion-positive solid tumors of 19 different histological subtypes, predominantly sarcomas, non-small cell lung cancer (NSCLC), and mammary analogue secretory carcinoma of the salivary gland. However, it has never been assessed in the treatment of thymomas. After 10 months of follow-up, the patient showed a significant response with mild adverse events, which was managed by temporary discontinuation of the drug. This case highlights the crucial role of whole-genome sequencing and tissue-agnostic antineoplastics in the future of cancer treatment.

## Introduction

Thymic neoplasms, a rare type of cancer, are estimated to have an annual incidence rate of only 0.13-0.15 per 100,000 individuals in the United States [1,2]. Based on their histological characteristics, they are classified into thymoma and thymic carcinomas. Thymomas have a more benign course and are usually confined to the thymus. A few studies have reported that thymomas might be driven by mutations in different genes, mainly epithelial growth factors and DNA repair genes [3]. Chromosomal rearrangements involving neurotrophic tyrosine receptor kinase (NTRK) genes are encountered in only 0.3% of all solid tumors and are associated with very rare histologic subtypes [4]. Until now, no cases of NTRK gene fusions in thymoma have been reported in the literature. In this report, we present a rare case of NTRK fusion-positive metastatic thymoma that showed a significant response to entrectinib, a novel NTRK-fusion inhibitor.

## Case presentation

**Figure 1:**
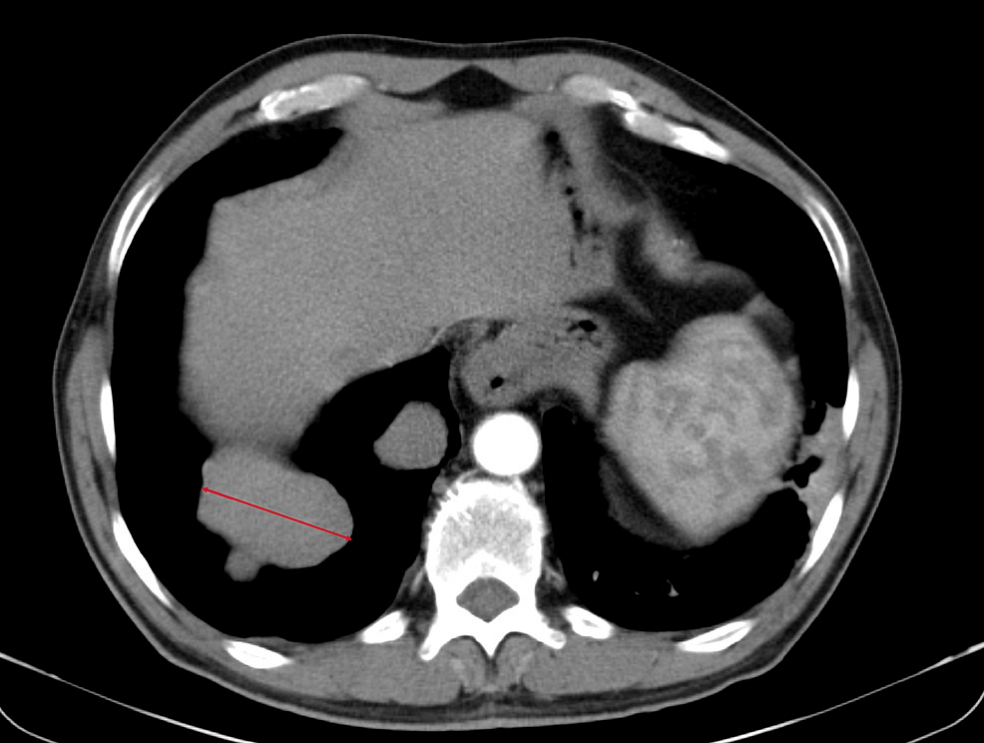
Lateral pleural lesion measuring 5.8 cm (arrow)

**Figure 2:**
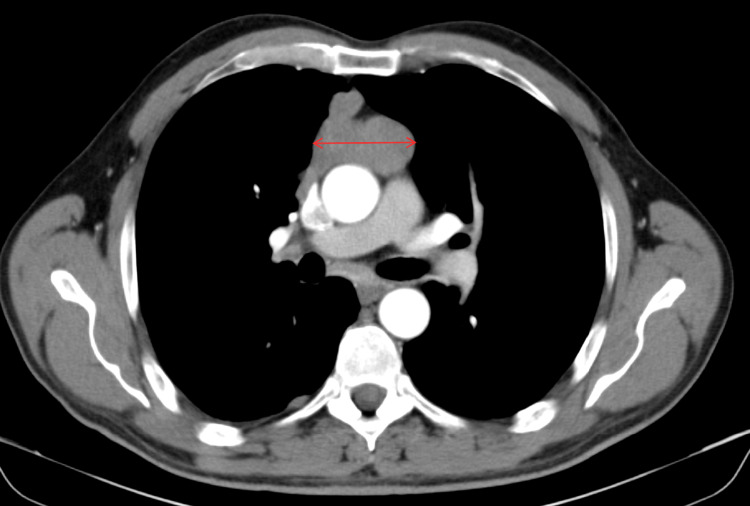
Mediastinal lesion measuring 5.2 cm (arrow)

**Figure 3:**
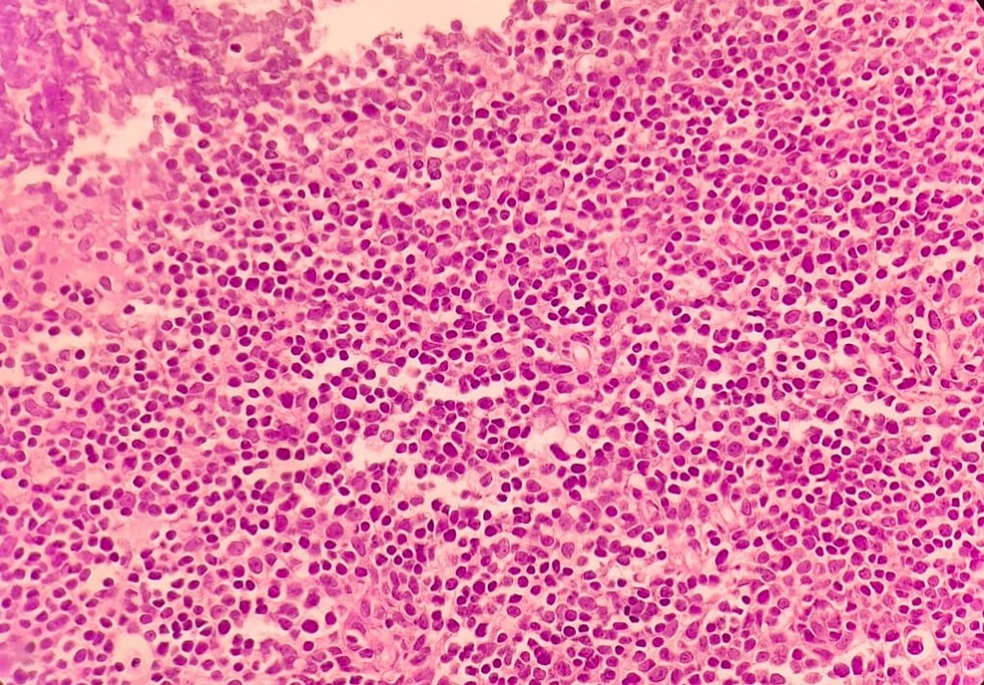
Light microscopy under H&E stain H&E: hematoxylin and eosin

**Figure 4:**
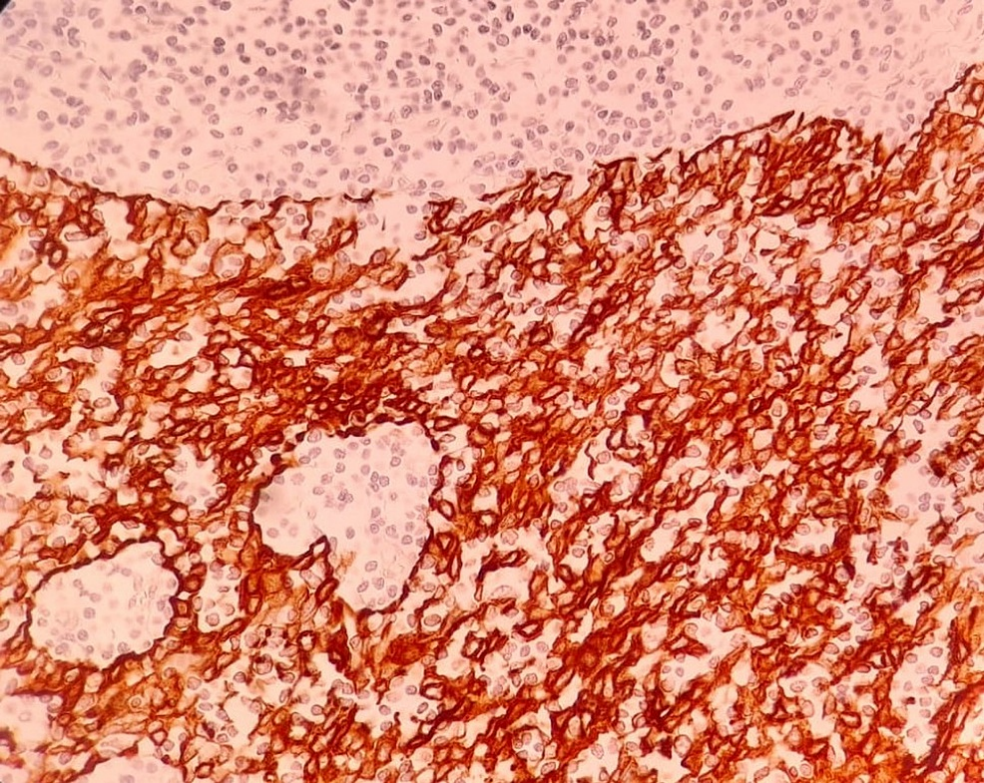
Immunohistochemical staining for cytokeratin AE1/AE3

Our patient was a 50-year-old male, with no significant past medical history, who presented to our university hospital with symptoms of vomiting and diffuse abdominal pain of several hours’ duration associated with moderate tenderness on the right lower quadrant on palpation, highly suspicious for appendicitis. An abdominal and pelvic CT scan showed no radiological signs of appendicitis but demonstrated the presence of multiple suspicious lesions on the right pleura. CT scan of the thorax showed multiple pleural-based masses in the right hemithorax, with the largest one located on the lateral right hemidiaphragm and measuring 5.8 cm (Figure 1). In addition, multiple masses were detected in the anterior mediastinum, the largest reaching 5.2 cm in size (Figure 2). The patient denied having any cough, dyspnea, chest pain, or any other symptoms concerning for space-occupying lesions in the thorax. At that time, he had an Eastern Cooperative Oncology Group (ECOG) performance status of 2. A CT scan-guided biopsy of the right lung mass demonstrated clusters of large epithelioid cells with mild atypia, intermixed with a dense population of small lymphoid cells (Figure 3). On immunohistochemistry (IHC) staining, cytokeratin AE1/AE3 (CKAE1/AE3) highlighted a complex meshwork of epithelial cells (Figure 4), and background lymphoid cells were positive for CD3, CD5, terminal deoxynucleotidyl transferase (TdT), and partially positive for CD10 and negative for CD20, CD30, CD34, and paired box protein-5 (PAX-5). These findings were deemed to be compatible with thymoma, which was confirmed by a second biopsy.

IHC stains showed that the tumor cells had a strong and diffuse membranous positivity for pantothenate kinase (PANK) and negativity for thyroid transcription factor 1 (TTF-1) while lymphoid cells were positive for CD45 and TdT. Therefore, the thymoma subtype B3 was confirmed. Based on the histological and radiological results, the patient was diagnosed with thymoma stage IV according to both Masaoka and TNM staging systems with multiple foci on the right diaphragmatic pleura and mediastinal lymph node involvement. Subsequently, he was started on cisplatin-based chemotherapy [CAP regimen: cyclophosphamide 500 mg/m^2^ intravenous (IV), doxorubicin 50 mg/m^2^ IV, and cisplatin 50 mg/m^2^ IV]. After two cycles of therapy, a follow-up CT scan showed a significant decrease in the size of lesions in the mediastinum and on the pleura abutting the right hemidiaphragm [partial response based on the Response Evaluation Criteria in Solid Tumors (RECIST) 1.1]. However, the patient expressed his refusal to continue chemotherapy treatment due to intolerable side effects. After extensive counseling, we decided to respect the patient’s wish to not pursue further treatment. Yet, a tissue specimen was sent for IHC staining and next-generation sequencing (NGS by Blueprint Genetics, Helsinki, Finland) to determine his eligibility for targeted therapies. While additional IHC staining confirmed a retained expression of mutL homolog 1 (MLH1), MutS homolog 2 (MSH2), MutS homolog 6 (MSH6), and postmeiotic segregation increased 2 (PMS2) and a low expression of programmed death-ligand 1 (PD-L1) on tumor cells, the NGS indicated an equivocal microsatellite instability, a high tumor mutational burden (31 m/MB), and the presence of tumor protein 53 (TP53) loss of function missense variant, fibroblast growth factor receptor 1 (FGFR1) copy number gain, and eukaryotic translation initiation factor 4B-neurotrophic receptor tyrosine kinase 3 (EIF4B-NTRK3) chromosomal rearrangement. No fms related receptor tyrosine kinase 1 (FLT1), fetal liver kinase 1/kinase insert domain receptor (FLK1/KDR), fms-related receptor tyrosine kinase 3 (FLT3), epidermal growth factor receptor (EGFR), or kit genes variants were identified.

**Figure 5:**
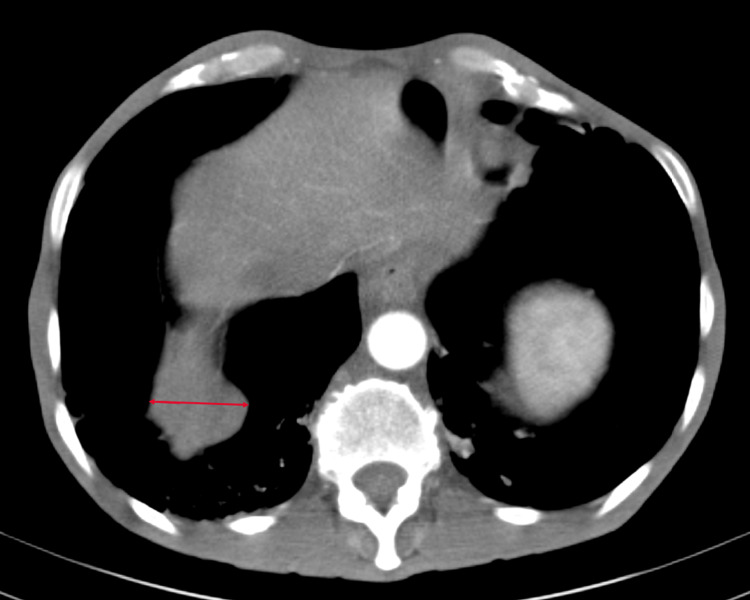
Lateral pleural lesion measuring 3.1 cm (arrow)

**Figure 6:**
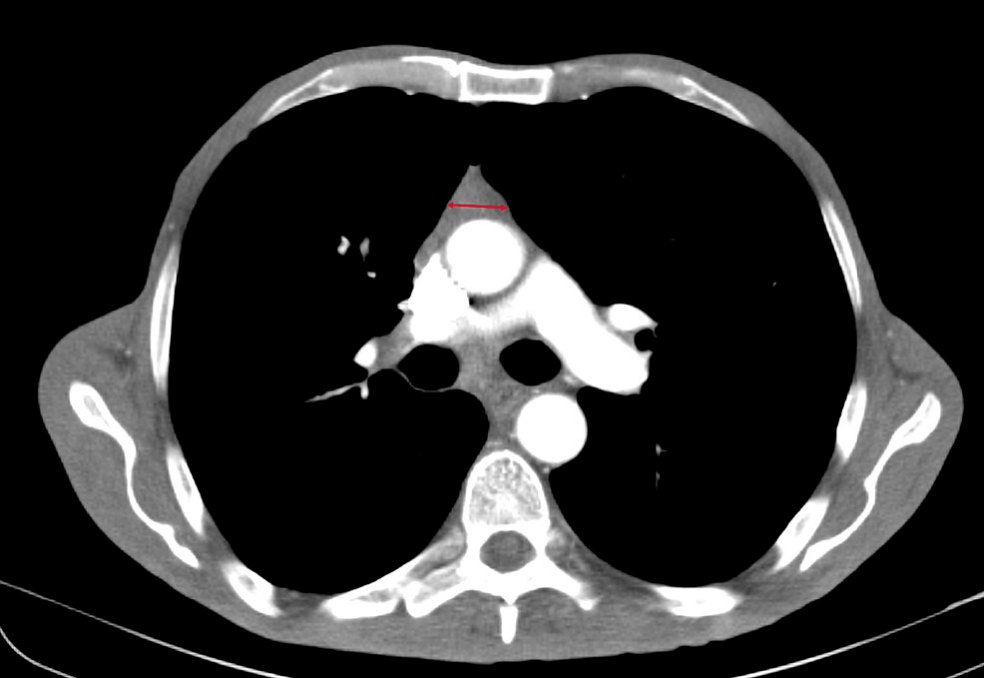
Mediastinal lesion measuring 1.3 cm (arrow)

After a careful review of these results, immunotherapy with anti-PD1 or anti-PD-L1 agents such as pembrolizumab was considered of low therapeutic benefit. However, the presence of chromosomal rearrangement involving the NTRK gene encouraged the use of entrectinib (ROZLYTREK™, Roche, Switzerland) which, at the time, had just been approved by the Food and Drug Administration (FDA) for use in NTRK fusion-positive solid tumors [5]. The consent to start treatment with entrectinib was obtained from the patient. Baseline echocardiography, EKG, and basic blood metabolic panel showed acceptable results for his age. Baseline chest CT scan demonstrated disease stability with no signs of progression after two cycles of chemotherapy. The patient was still asymptomatic and had preserved performance status. Entrectinib at the dose of 600 mg tablet PO once daily was then initiated. The patient had grade 2 diarrhea during the first few weeks of treatment initiation, which subsided without medical intervention. He was followed up closely with regular physical examination, while radiological exams were not done due to the coronavirus disease 2019 (COVID-19) pandemic. However, 10 months after starting the drug, he presented to the emergency department with a decreased level of consciousness, generalized muscle weakness, profuse watery diarrhea, and a diffuse psoriasis-like skin rash of gradual onset of over one month. After extensive workup, myasthenic crisis was confirmed with the involvement of the extraocular, respiratory, and skeletal muscles. Appropriate treatment with steroids and pyridostigmine was then started. In addition, as per the guidance provided by the drug manufacturer for the management of adverse effects, the drug was withheld. The patient was monitored closely in the hospital until the resolution of the neurological symptoms and was then discharged after the improvement in his respiratory function. One month later, the drug was restarted successfully with no new reported adverse events. A control chest CT scan performed after 10 months of treatment with entrectinib demonstrated a confirmed partial response (35% reduction in the sum of the longest diameters, RECIST 1.1) (Figures 5, 6).

## Discussion

Thymomas are much rarer than thymic carcinomas. Several studies have reported that roughly 90% of patients had, at presentation, a disease stage equal to or lower than stage III (based on either Masaoka or TNM staging systems) while stage IV disease at diagnosis was found in about 10% of patients [3]. Therefore, due to the rarity of thymomas, the list of differential diagnoses was particularly long in our case, including primary lung carcinoma, thymic carcinoma, or a metastatic spread of a distant tumor. Primary lung carcinoma was ruled out by TTF-1 negativity, while metastatic carcinoma was deemed unlikely since epithelial cells did not express severe atypia to be consistent with carcinoma. Moreover, thymic carcinoma was excluded due to the mild level of atypia as well as the presence of immature TdT+ T-lymphocytes, which are, respectively, major and minor criteria for the histologic diagnosis of thymic carcinoma, based on the WHO histological classification of thymic cancers [6]. Thymomas are further classified into histologic subtypes A, AB, B1, B2, and B3 according to the predominant cell type and morphology. In this case, the B3 subtype was diagnosed based on the predominance of epithelial cells compared to the lymphoid cells (less than 50%). Despite being considered to be less aggressive than thymic carcinomas, the B3 subtype has a moderate to high malignant potential, which is consistent with the advanced stage at presentation in the case of our patient [7]. The primary tumor was not assessed as more accessible pleural-based lesions were confirmed on histology to be thymoma, obviating the need for further invasive procedures.

Most patients with thymoma have symptoms at presentation, which can include those caused by tumor impingement on surrounding tissues (40%) such as chest pain, cough, hoarseness, dyspnea, and systemic symptoms such as weight loss, fever, and night sweats (30%). Interestingly, one-third of patients are asymptomatic at initial presentation, similar to our patient. Paraneoplastic syndromes are also common with thymomas, specifically myasthenia gravis, which occurs in approximately 30-45% of patients with thymomas [3,7]. Myasthenia gravis initially affects ocular muscles in 50% of cases and more than half of those will develop a generalized disease within two years. Pathogenesis of thymoma-associated myasthenia gravis is complex and still not fully elucidated, but it is believed that thymomas of subtypes B have a stronger association with myasthenia gravis than those of subtypes A and AB [7]. Our patient presented with a severe case of myasthenic crisis probably due to the delay in seeking medical care. The diagnosis was based on the clinical presentation and was confirmed by the characteristic findings on electromyogram and nerve conduction studies as well as the serologic evidence of antibodies against acetylcholine receptors. Response to treatment was rapid with the recovery of muscular function within two to three weeks.

The initial management of thymomas depends on the disease stage with surgery being the cornerstone of the therapeutic strategy for operable disease, mainly in stages I-II as well as selected cases of stages III and IV. Postoperative survival depends on the stage, the completeness of resection, and the histological subtype. When radical resection is not an option in locally advanced and metastatic disease, multimodality therapy is the mainstay of treatment, consisting of chemotherapy and/or radiotherapy [8]. In recent years, several systemic treatments have been assessed in thymomas with varying response rates depending on the tumor and patients’ features. The CAP regimen was employed in our patient as it is one of the preferred regimens for metastatic thymoma IVb [9]. Two cycles were administered, which induced a partial response but was associated with significant adverse effects deemed intolerable by the patient. Yet, we were able to convince him to explore second-line options, specifically targeted therapies available in oral formulations.

Recently, targeting molecular and genetic alterations within the tumors has gained momentum in the management of different types of tumors. Chromosomal and genetic abnormalities, often implicated in the pathogenesis of cancer, have driven this trend toward the development of new therapeutic agents targeting specific signaling pathways within tumor cells. Thymic cancers were investigated by several studies for their expression levels of different growth factor receptors. These studies have found that some thymomas can overexpress epidermal growth factor receptor (HER-1 and HER-2), stem cell factor receptor (SCFR/KIT/CD117), insulin-like growth factor-1 receptor (IGF-1 R), vascular endothelial growth factor receptor (VEGFR), rat sarcoma proteins (RAS), and PD-L1, among others [3,10]. The implication of these molecular aberrancies is not yet well elicited. The evidence regarding the clinical response of thymic cancers to these innovative agents is mainly anecdotal except for the significant survival benefit derived from the use of pembrolizumab and sunitinib, which are respectively a PD-L1 inhibitor and multi-kinase inhibitor. The results of these trials have led to the inclusion of these two drugs to the list of second-line systemic therapy options for thymic cancers that are refractory to cytotoxic chemotherapy [10,11]. However, immunohistochemical and genetic-profiling results in our patient as well as concerns about higher rates of immune-related adverse effects reported in patients with thymomas treated with pembrolizumab encouraged us to investigate different options [12]. Furthermore, the low response rate (6%) reported in thymomas treated with sunitinib was not encouraging [11].

Nevertheless, the NGS results showed an EIF4B-NTRK3 chromosomal arrangement, which has put forward an unexpected therapeutic option by NTRK-fusion inhibitors. NTRK 1, 2, and 3 are genes that encode a family of neurotrophin receptors called tropomyosin receptor kinases (TrK-A, TrK-B, and TrK-C). These receptors are primarily expressed in neural tissues and play a major role in the growth regulation of these tissues [13]. Fusions affecting these genes result in TrK with constitutively active kinase function and their frequency is estimated to be about 0.3% of all solid tumors. Moreover, thymic cancers are not known to be among the rare malignant entities enriched for NTRK-fusions, and hence no case of thymic cancer was recruited in the clinical trials that have evaluated the efficacy of NTRK-fusion inhibitors [4,13]. This highlights the extreme rarity of the mutation identified in this case of advanced thymoma.

Worldwide, two NTRK-fusion inhibitors have been approved so far for NTRK fusion-positive cancers: larotrectinib (VITRAKVI™) and entrectinib (ROZLYTREK™). Entrectinib is a multi-kinase inhibitor with strong activity against TrK and other kinases. Its efficacy in NTRK fusion-positive solid tumors was demonstrated in three single-arm, open-label phase 1 and phase 2 clinical trials (ALKA, STARTRK-1, and STARTRK-2). These trials enrolled patients with various solid tumor types, most commonly sarcoma (24%), lung cancer (19%), salivary gland tumors (13%), breast cancer (11%), thyroid cancer (9%), and colorectal cancer (7%). The overall response rate was 57% (95% CI: 43-71) including 7% of complete responses while 45% of patients achieved durable responses of more than a year [4]. Treatment was well tolerated by the patient discussed in this paper except for a non-serious, grade 3 adverse events (skin rash and watery diarrhea) that necessitated the temporary discontinuation of the drug for one month, after which it was safely restarted with no recurrence of the symptoms. It is not clear whether the emergence of myasthenia gravis is related to the treatment with entrectinib; however, a strong association between thymomas and myasthenia gravis has been previously reported. The results of the chest CT scan at 10 months post-initiation of entrectinib have demonstrated a partial radiological response with clinical improvement. This result is considered to be remarkable in stage IVb thymoma B3, which is commonly categorized as a high-risk thymoma [7], thereby highlighting the importance of implementing molecular and genetic testing in the management of rare tumors.

## Conclusions

This case report highlights the new therapeutic options that are offered by precision medicine to patients with previously difficult-to-treat rare cancers. It also emphasizes the relevance of broadening the application of NGS and other genetic-profiling methods adopted by precision medicine in identifying potentially actionable genetic variants. Our experience with entrectinib confirms the robust and durable response seen in the trials conducted on the path to its approval. Furthermore, by reporting the successful use of entrectinib in treating this case, the authors hope to contribute to advancing the new trend toward developing new tissue-agnostic antineoplastics that have a broad scale of applications.
